# A Research‐inspired biochemistry laboratory module–combining expression, purification, crystallization, structure‐solving, and characterization of a flavodoxin‐like protein

**DOI:** 10.1002/bmb.21218

**Published:** 2019-02-11

**Authors:** Marta Hammerstad, Åsmund K. Røhr, Hans‐Petter Hersleth

**Affiliations:** ^1^ Department of Biosciences, Section for Biochemistry and Molecular Biology University of Oslo NO‐0316 Oslo Norway; ^2^ Department of Chemistry, Biotechnology and Food Science Norwegian University of Life Sciences NO‐1432 Ås Norway; ^3^ Department of Chemistry, Section for Chemical Life Sciences University of Oslo NO‐0315 Oslo Norway

**Keywords:** Biochemistry, structural biology, laboratory exercises, research‐inspired, project‐based, proteins, crystallography, spectroscopy, protein, purification, protein chemistry

## Abstract

Many laboratory courses consist of short and seemingly unconnected individual laboratory exercises. To increase the course consistency, relevance, and student engagement, we have developed a research‐inspired and project‐based module, “From Gene to Structure and Function”. This 2.5‐week full‐day biochemistry and structural biology module covers protein expression, purification, structure solving, and characterization. The module is centered around the flavodoxin‐like protein NrdI, involved in the activation of the bacterial ribonucleotide reductase enzyme system. Through an in‐depth focus on one specific protein, the students will learn the basic laboratory skills needed in order to generate a broader knowledge and breadth within the field. With respect to generic skills, the students report their findings as a scientific article, with the aim to learn to present concise research results and write scientific papers. The current research‐inspired project has the potential of being further developed into a more discovery‐driven project and extended to include other molecular biological techniques or biochemical/biophysical characterizations. In student evaluations, this research‐inspired laboratory course has received very high ratings and been highly appreciated, where the students have gained research experience for more independent future work in the laboratory. © 2019 The Authors. *Biochemistry and Molecular Biology Education* published by Wiley Periodicals, Inc. on behalf of International Union of Biochemistry and Molecular Biology, 47(3):318–332, 2019.

## Introduction

Common to many laboratory courses is the organization of the topics into shorter and seemingly unconnected or fragmented individual laboratory exercises. Each exercise focuses on learning a set of skills or understanding a concept or approach where a certain biochemical system is chosen, while another system is chosen for the next exercise. It should, however be considered, if more relevant approaches applicable to actual research projects and procedures could trigger student engagement and increase their knowledge and skills.

It has been pointed out that an approach with individual laboratory exercises leaves the students lacking an understanding of the big picture 1. The students often lack the understanding of how the skills they learn in the different laboratory exercises are related, and how they are used together in a real research environment. There are several recent examples of undergraduate biochemistry laboratory courses that have succesfully implemented a more integrated and research‐inspired approach 1, 2, 3, 4. These courses cover different aspects of the biochemistry education and have different focuses with respect to topics, length, class size, and use of teaching resources. The course presented in this article focuses on the structural and functional aspects of a given redox protein, designed as a 2.5‐week intensive course but could also be implemented as a semester‐based course run once a week for 10–14 weeks. Although several of the above mentioned laboratory courses cover purification and characterization of a chosen model protein, our course covers a unique combination of topics including protein expression and purification, crystallization, structure solving and analysis, and spectroscopic characterization of a redox protein, which is part of a novel enzymatic system.

### The Ribonucleotide Reductase System and the NrdI Protein

**Figure 1:**
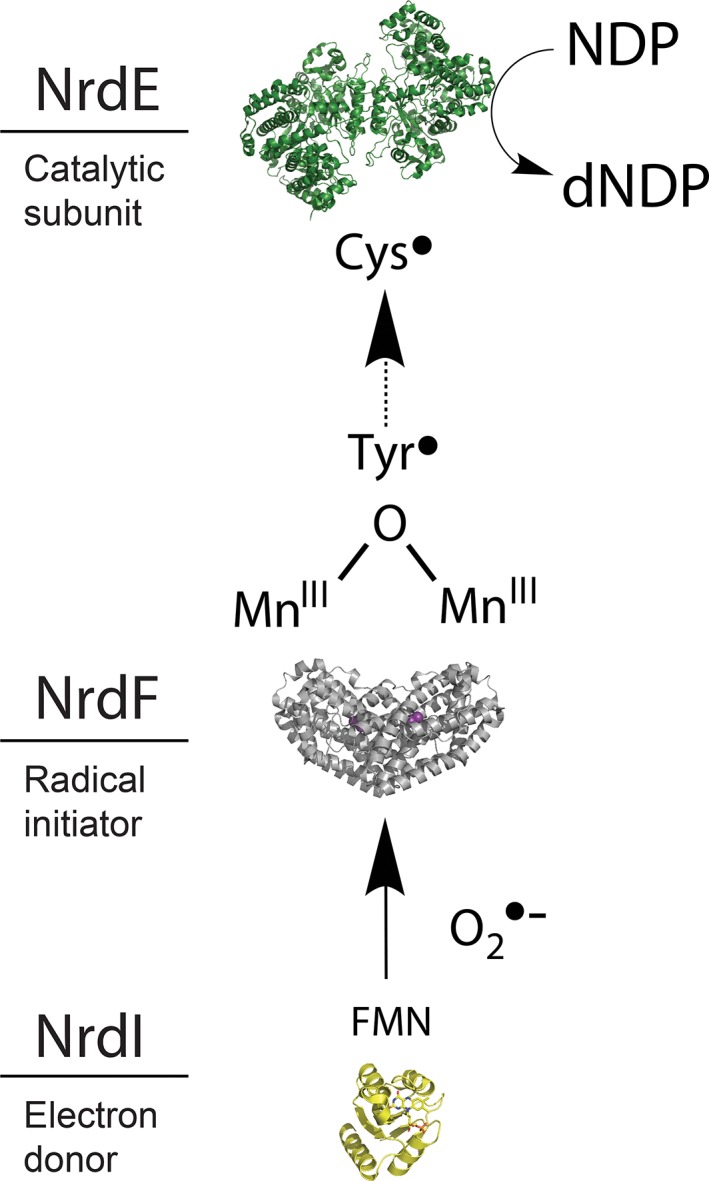
Schematic overview of the RNR class Ib activation pathway including the flavoprotein NrdI, the radical‐initiating subunit NrdF, and the catalytic subunit NrdE. The FMN cofactor in NrdI is represented as sticks and colored by atom type. The manganese ions in NrdF are shown as purple spheres. [Color figure can be viewed at wileyonlinelibrary.com]

The “From Gene to Structure and Function” module is designed as a study of one specific protein, NrdI. NrdI is a 119 amino acid residue flavodoxin‐like protein that is found in the ribonucleotide reductase (RNR) operon in *Bacillus cereus*. RNRs catalyze the reduction of ribonucleotides (NDPs or NTPs) to their corresponding deoxynucleoside 5′–di– or triphosphates (dNDPs or dNTPs), and hence, play an important role in nucleotide metabolism in all DNA‐based living organisms 5. All RNRs share a common catalytic mechanism involving the activation of a ribonucleotide by abstraction of the 3′‐hydrogen atom of ribose by a transient thiyl radical (Cys•) in the catalytic subunit, leading to the exchange of the hydroxyl group on the 2′‐carbon of the ribose ring with a hydrogen atom 6 (Fig. 1). RNRs are divided into three main classes, differing in how they generate the Cys•. The aerobic bacterial class Ib RNR subunits are encoded by the *nrdE* and *nrdF* genes, encoding the catalytic‐ and radical‐activating subunits, respectively 7. However, the class Ib RNR operon structure encodes two additional proteins; NrdH and NrdI, organized in a co‐transcribed *nrdHIEF* operon structure 8.

**Figure 2:**
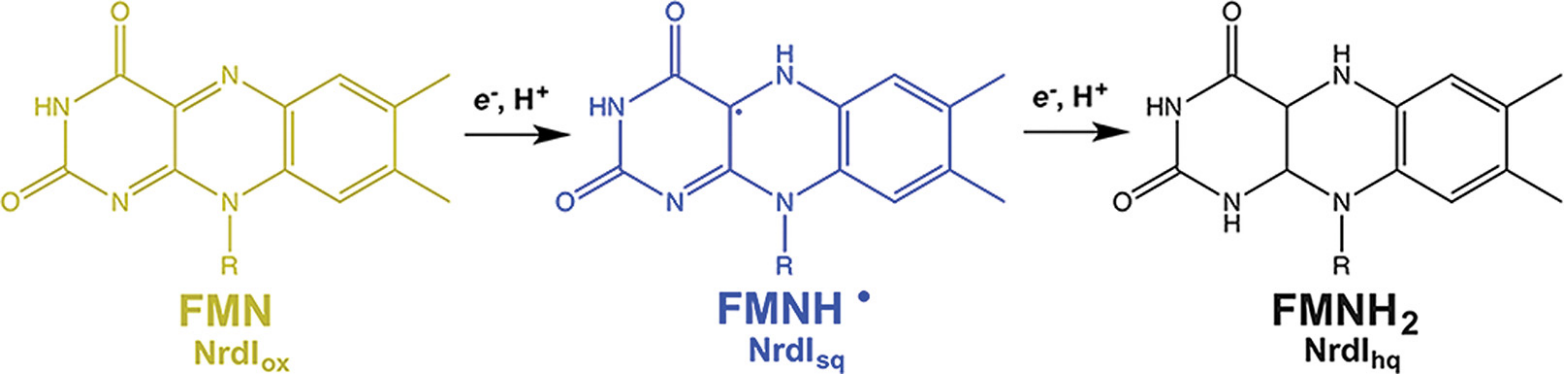
The three main oxidation states of the FMN cofactor in NrdI. The cofactor is reduced and protonated step‐wise, with each step causing a change in absorption of UV–visible light and, hence, change in color (FMN, yellow; FMN^•^, blue; FMNH_2_, colorless). [Color figure can be viewed at wileyonlinelibrary.com]

**Figure 3:**
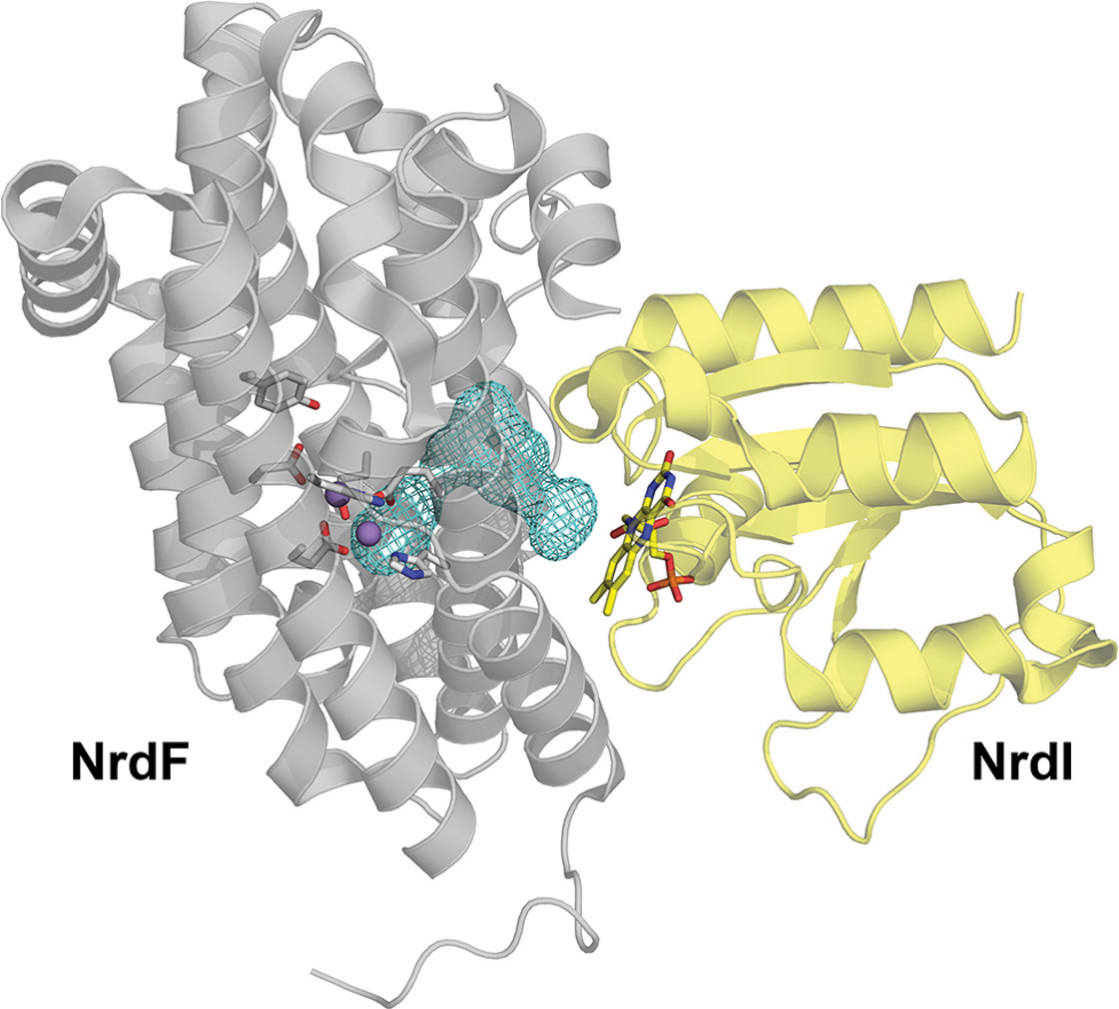
Structure of the class Ib RNR NrdI‐NrdF complex from Escherichia coli (PDBid:3N39) 12, showing the solvent channel (mesh) connecting the FMN cofactor in NrdI with the di‐manganese cluster in NrdF. The FMN cofactor, as well as the residues on the active site of NrdF are shown as sticks. Manganese ions are shown as spheres. [Color figure can be viewed at wileyonlinelibrary.com]

Class Ib RNR utilizes an active Mn^III^_2_‐tyrosyl radical (Y•) cofactor in the NrdF subunit for the generation of the transient Cys• in the catalytic site of NrdE. Contrary to the self‐assembly of the active Fe^III^_2_‐Y• cofactor in the class Ia RNRs, the active Mn^III^_2_‐Y• cofactor in class Ib RNR can only be generated from the Mn^II^_2_ site in the presence of O_2_ and the reduced flavodoxin‐like protein NrdI (see Fig. 1). NrdI, encoded by the *nrdI* gene, is found in all organisms with genomes coding for the class Ib RNR 9, 10. NrdI contains an FMN cofactor, demonstrated to be able to act as an electron donor. The structures of the three observable oxidation states of the FMN cofactor (FMN/FMNH•/FMNH_2_) in NrdI, are shown in Fig. 2. Studies by Stubbe and coworkers have suggested that the fully reduced hydroquinone form of NrdI (NrdI_hq_, containing FMNH_2_) activates O_2_ and produces a reactive oxygen in a reaction that oxidizes NrdI to its neutral semiquinone form (NrdI_sq_, containing FMNH•) 11. The activated oxygen species is believed to diffuse through a proposed hydrophilic solvent channel 12, 13, extending from the flavin cofactor of NrdI to metal site 2 in NrdF, ultimately generating the active Mn^III^_2_‐Y• cofactor in class Ib RNR (Fig. 3).

### The Ideas behind the Course

The biochemistry laboratory course described in this article was originally taught as individual laboratory exercises, but was redesigned in 2010 to include the research‐inspired module involving studies of the NrdI protein to increase the course consistency, relevance, and student engagement. The module entitled “From Gene to Structure and Function,” focused on structural biology, was developed and has repeatedly been optimized and taught in total 13 times. This course is part of a molecular bioscience laboratory course series, intended to give new master students in molecular biosciences a toolbox and a general knowledge of important techniques within the field useful for the students' future research or work both within or outside academia.

The focus of this module is to cover methods and techniques in biochemistry and structural biology related to protein expression, purification, crystallization, structure solving with protein crystallography and characterization with spectroscopy. We have developed a project that covers all these methods and that is research‐inspired. Therefore, the project was developed so that the students would study and work with one protein through all the steps. By following the same protein through the whole module, we intended to give the students a stronger relation to the project and the laboratory course. This should lead the students to better understand the link between the different methods and their importance in the study of proteins. To further enhance the idea of seeing the project as a bigger picture, the students write a report for the whole project as an article, where they combine all the results in a comprehensive and scientific way.

By going in depth in studying one given protein, the students gain experience and knowledge in order to be able to broaden the use of these methods for other problems and proteins in the future. The importance of an in‐depth approach has been noted in science education 14, however, the focus has to be such that the depth opens up for a broad understanding. To support this, some additional lectures, tutorials, and exercises have been included in the module.

When developing the course, it was also important to make it relevant and research inspired. Therefore, the NrdI protein involved in the RNR system, which is an important and active research field, was choosen. This also allows students to benefit from original research, and the idea of contributing to an active research field 15. When designing the course, we aimed to create a balance between the degree of how discovery‐based versus how closely‐guided the course should be. This meaning, constructing a course with a suitable level of directions provided by the instructors, and allowing for a certain degree of student variation and reasoning, the latter shown to be valuable in growing the students competence to engage in science 16, 17. To generate a robust course that can be carried out with high reproducibility and that can easily be run for classes of 30–40 students (or larger) without the need of too many teaching assistants (TAs), the course requires a certain degree of guidance. Therfore, it is also important that the course is encouraging and question driven in a research‐inspired way. The students are asked to answer scientific questions about the function and structure of the protein they are studying as a driving force and guided through the laboratory course to be able to answer these questions. It is possible to redesign the course slightly to open up for more discovery‐based teaching in a course‐based undergraduate research experiences (CUREs) manner 16, as will be described below.

### Overview of the Course

The workload of the module equals to five credits in the European Credit Transfer and Accumulation System. It is obligatory for master students in molecular biology and biochemistry at our department, and most master students attend the course during their first fall semester. The module is given intensively over 2.5 weeks, with full‐day teaching, where any periods of time between experimental work in the laboratory is filled with lectures, computer laboratory, or student exercises. The lectures/exercises provide a survey of the experimental methods used during the course, giving students the needed technical background, and have a focus on active student learning. Some study periods are scheduled in between, as some laboratory group sessions are run in parallell. During the practical parts of this course, students work in pairs, or at a maximum of three students per group, depending on the number of participating students during different semesters, while in the computer laboratory, they work individually. Course enrollment has varied from 10 to 42 students but is suited for smaller or larger groups, depending on the availability of resources at a given department. In addition to the main teacher responsible for the course, an assigned group of TAs is involved in the implementation of the course. Typically, a ratio of one faculty and four TAs per 40 students is engaged throughout the period. The TAs include PhD students and postdoctoral researchers with general biochemical backgrounds as well as some with structural biological expertise. Depending on the TAs previous knowledge of the techniques used, a 1–5 day training is given, in addition to self‐studying of provided technique background.

During the 2.5‐week period, each student will have 25 hours wet laboratory, 14 hours computer laboratory, 18 hours lectures, and 12 hours exercises. All experimental protocols have been thoroughly optimized in order to gain a high protein yield with the highest purification grade, during a short period of time. The course is partly run in the teaching laboratory and in research laboratories at the department. The use of research laboratory instrumentation, for example, for protein purification and spectroscopic characterization further increase the research aspect of the course.

### Benefits of Using this Protein

The NrdI protein has shown to be a well‐suited model protein for such a laboratory course, with several advantages. The protein is highly overexpressed at 30°C during a timespan of approximately 24 hours, with a very low degree of protein aggregation. Also, the protein has shown high stability when kept on ice, but also to a large extent at room temperature, showing no or very limited precipitation and loss in yield. Importantly, the presence of an FMN cofactor is a great advantage for several reasons; making the protein easy to visualize and detect during protein handling including protein extraction and protein chromatography; providing a basis for studying redox properties with UV–vis spectroscopy; and visualization of bright, yellow‐colored crystals. The small size of 13.5 kDa contributes to the ease of purification, leading to a satisfactory purity requirement, which has always assured successful crystal growth. A small protein size is also convenient for a comprehensive refinement and analysis of the protein structure performed by the students, given the time frame of the module.

## Teaching and Experimental Procedures

**Figure 4:**
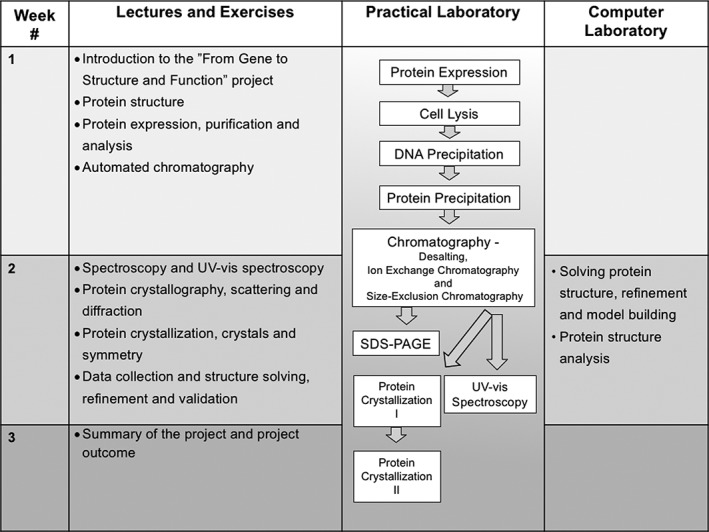
Overview of the “From Gene to Structure and Function” laboratory and teaching timeline.

As a prerequisite for taking this course, students have gained knowledge in the field of recombinant DNA technology and its applications through another course held at the department. The preceding course covers topics such as gene cloning, methods for mutagenizing DNA, bioinformatics tools for managing and analyzing DNA and protein sequence, and methods for analysis of gene expression. The laboratory course presented in this article can be seen as an extension of the latter applications, involving a deeper understanding of the structural and functional aspects of a given protein. A summary of the laboratory and teaching module timeline is shown in Fig. 4.

To increase the students' engagement and the feeling that the project is relevant and research‐based, we ask the students to answer two main questions about the NrdI protein. The first question is whether the NrdI protein in *B. cereus* can act as a one‐ or a two‐electron donor. Originally, it was suggested that NrdI in *Escherichia coli* can serve as a two‐electron donor 9. In the latter case, hydrogen peroxide will be generated as the reactive oxygen species; however, if the protein acts as a one‐electron donor, superoxide will be generated. One way of answering this question is to perform redox titration of the NrdI protein and observe the reduction by UV–vis spectroscopy, as the flavin cofactor changes color and spectroscopic properties between different redox states (Fig. 3). If the blue semiquionone state builds up, the redox potentials between the two redox couples (FMN_ox_/FMN_sq_ and FMN_sq_/FMN_hq_) must be different, and hence, the protein can function as a one‐electron transporter. The second question the students must answer is what structural changes occur in the vicinity of the flavin cofactor when it changes redox state. The answer can be found by solving the structure of the protein in different redox states and comparing both structures. Therefore, to be able to answer these questions during the course, the students must express and purify the protein, in order to perform a redox titration of the protein, crystallize the protein, and solve and interpret the structure. Due to time constraints, all solutions used in the course are prepared in advance by the TAs or laboratory technicians.

### Part 1: Protein Expression

#### *Background*

In addition to the students' prior knowledge in DNA technology, this course introduces and covers further knowledge regarding various protein expression systems, considerations during protein production, and vector choice. The course also gives an introduction to the T7 expression system used to express the recombinant NrdI protein in *E. coli* during this course.

#### *Experimental*

The gene encoding *B. cereus* NrdI (Locus tag BC1353) has previously been inserted into the pET‐22b(+) vector using restriction sites XbaI and HindIII. This is a vector used in the T7 *E. coli* expression system. The plasmid harboring the *nrdI* gene was transformed into chemically competent BL21(DE3) *E. coli* cells (Stratagene) prior to the course.

On Day 1, students start growing the overnight cultures. On the afternoon, each group of students inoculate a sterile 250 mL Erlenmeyer flask containing 50 mL Luria broth medium supplemented with 100 μg/mL ampicillin with a single colony picked from an agar plate preincubated with NrdI‐expressing *E. coli* cells. The flasks are incubated overnight at 30°C in a shaking incubator.

On the morning of Day 2, each group transfers its overnight culture to a sterile 2 L baffled Erlenmeyer flask containing 1 L Terrific Broth (TB) medium supplemented with 100 μg/mL ampicillin. The cultures are further incubated in a rotary shaker for 2 hours at 30°C (OD_600nm_ = 0.7–0.9). Prior to induction, 500 μL culture is collected in eppendorf tubes from each culture flask, and the sedimented bacteria are stored at – 20°C for later sodium dodecyl sulfate polyacrylamide gel electrophoresis (SDS‐PAGE) analysis. Protein expression is induced through the addition of isopropyl β‐D‐1‐thiogalactopyranoside to a final concentration of 1 mM in each culture flask, and the cultures are further incubated in a rotary shaker for 5 hours at 30°C. Prior to cell harvesting, another culture sample of 200 μL is collected by each group for later SDS‐PAGE analysis. The cells are harvested in the afternoon of Day 2, in 500 mL centrifuge cups by spinning for 8 minutes at 5,000 x g using a JA10 rotor, transferred to zip‐locked plastic bags, and stored at –20°C.

#### *Results*

A typical yield of wet bacterial paste stored in zip‐locked bags, resulting from 12 L TB medium expressing the NrdI protein is 90–180 g. Depending on the amount of students attending the course, 30–60 g of bacterial paste is used for further cell lysis. The degree of protein overexpression is analyzed by SDS‐PAGE.

### Part 2: Cell Lysis and Protein Extraction

#### *Background*

The method of choice for bacterial cell‐wall disruption in this course is sonication, which efficiently lyses the bacteria by applying high‐frequency energy to agitate and disrupt the cells. The course also gives a thorough introduction to protein precipitation (salting‐out) as the method of choice for protein extraction, but also as the first, and crude protein purification step.

#### *Experimental*

On Day 3, 30–60 g of cells are dissolved in 50 mM Tris–HCl, pH 7.5, 5 μg/mL DNase, protein inhibitor cocktail tablet (Roche), in a 1:4 ratio of cell wet weight‐to‐buffer volume. The teacher demonstrates the cell lysis by sonicating the cell suspension in 60 mL batches at 50% amplitude, with three bursts of 20 seconds followed by intervals of 40 seconds for cooling on ice. Each group recieves 20–25 mL of lysed cell suspension.

The samples are from now on maintained on ice, and vigorious stirring is avoided. Particulate matter is removed from the sample by centrifuging in 50 mL tubes at 48,000 x g, 4°C, for 45 minutes using a JA25.50 rotor. The students note the volume of the supernatant and keep it for the next step.

Addition of streptomycin sulfate to a final concentration of 2% (w/v) effectively precipitates DNA, leaving proteins in solution. While carefully stirring, students add a solution of 10% streptomycin sulfate corresponding to one‐fourth of the volume of their supernatant, dropwise. The samples are then centrifuged in 50 mL centrifuge tubes for 15 minutes at 27,000 x g, 4°C, using a JA25.50 rotor. The resulting volume of the supernatant is measured for the next step of protein precipitation.

Ammonium sulfate ((NH_4_)_2_SO_4_) is typically used to “salt out” proteins from solution. In order to acchieve a high protein yield, students add solid ammonium sulfate to a final concentration of 0.43 g/mL in small amounts, while stirring their protein solutions carefully. The solutions are left to equilibrate while stirring for 10 minutes. Each student group sample is then centrifuged for 15 minutes at 27,000 x g, 4°C, using a JA25.50 rotor, the supernatant is discarded, and the protein precipitate is carefully dissolved in 3 mL 50 mM Tris–HCl, pH = 7.5, giving a total volume of approximately 5 mL protein solution.

### Part 3: Protein Chromatography

#### *Background*

The course covers different strategies for protein purification, based on proteins various physio‐chemical properties, such as charge, polarity, size and specific ligand binding. The students get particular knowledge in ion‐exchange chromatography (IEX) and size‐exclusion chromatography (SEC), as these chromatographic methods are also performed in the practical laboratory of this course. Each group gets assigned to perform fast protein liquid chromatography (FPLC) for the IEX and SEC chromatograpic procedures. In this course, we use the ÄKTA FPLC purification system (GE Healthcare, Oslo, Norway). The students will be instructed in operating the system, constructing suitable programs, and analyzing the chromatographic data. As the flavoprotein NrdI contains an FMN cofactor, the protein is yellow in its completely oxidized form (NrdI_ox_), and exhibits a UV–vis absorption maximum at ≈ 450 nm. This feature is used to identify NrdI from other proteins in the chromatographic analysis, making the purification process highly convenient and instructional.

#### *Experimental*

##### *Desalting by SEC*

Due to the ease of optimization of this step, the desalting of NrdI (neccessary to enable protein binding to the IEX chromatography resin) is performed by each student group on the laboratory bench.

The dissolved protein from the ammonium sulfate precipitation step is clarified by a 5 mL syringe and a 0.45 μm syringe filter (Sarstedt). Each group is provided with a 5 mL HiTrap Desalting column (GE Healthcare), which they equilibrate with 20 mL 50 mM Tris–HCl, pH = 7.5.

The protein solution is desalted in several steps, by applying 1.5 mL protein on the column, eluting the protein with 2 mL 50 mM Tris–HCl, pH = 7.5, followed by a re‐equilibration with 10 mL 50 mM Tris–HCl, pH = 7.5. These steps are repeated until the whole protein sample has been desalted. To ensure an adequate desalting of the samples, it is important to follow the procedure precisely. The desalted sample is stored for IEX, in addition to a 4 μL sample for later SDS‐PAGE analysis. If desirable, desalting may also be performed in an FPLC purification system.

##### *Anion‐Exchange Chromatography*

The next purification steps are carried out using the Äkta chromatography system. 90 minutes are typically scheduled for each group for the IEX procedure.

The desalted protein sample is clarified using a 0.2 μm syringe filter (Sarstedt), and the total sample from each group is applied on to a 50 mL Superloop (GE Healthcare) and the 5 mL Q Sepharose High Performance column (HiTrap Q HP) (GE Healthcare) mounted on the Äkta purifier. The protein is chromatographed in 50 mM Tris–HCl, pH = 7.5, and eluted using a linear gradient of 50 mM Tris–HCl, pH = 7.5, 1 M KCl (elution buffer). A suitable program for the automated process is constructed together with the teacher, and the process is followed and discussed. The pooled fractions containing the yellow NrdI protein (≈ 6–9 mL) are concentrated using a 15 mL Amicon Ultra centrifugal filter (Merck Millipore, Oslo, Norway) with a nominal molecular weight limit of 10 kDa, in a JA25.50 rotor at 5,000 x g, 4°C, for 50 minutes. The final concentrated sample is stored for SEC, in addition to a 4 μL sample for later SDS‐PAGE analysis.

##### *Gel Filtration by SEC*

Typically, 60 minutes are scheduled for each group for the SEC procedure.

At first, the anion‐exchanged protein sample is clarified using a 0.22 μm Ultrafree®‐MC (0.5 mL) centrifugal filter unit (Merck Millipore). Using a Hamilton syringe, 120 μL of the concentrated sample is then applied on a 500 μL loop mounted on the Äkta system. Several high‐resolution SEC columns have been succesfully used in the final polishing step for purification of the NrdI protein, including the Superdex 200 10/300 GL and the Superose 12 10/300 GL (GE Healthcare). The protein is eluted in 50 mM Tris–HCl, pH = 7.5. An appropriate program is constructed together with a teacher, and the protein is detected in the eluate using UV–vis absorption at 280 and 450 nm. The fractions are collected and concentrated in Amicon Ultra 15 mL and 0.5 mL centrifugal filters (Merck Millipore) with a 10 kDa pore size, to a final volume of approximately 50 μL pure NrdI protein. A 4 μL sample is frozen and stored for later SDS‐PAGE analysis.

#### *Results*

After the desalting step, each group acquires typically 4–7 mL of protein solution, depending on the success rate. The desalting step is important in terms of removing residual salt from the sample prior to applying it on the IEX column. Inadequately desalted samples may not bind to the IEX column. In such cases, groups are encouraged to troubleshoot and discuss the likelihood of improved binding to the column either by a subsequent desalting step or by increasing the pH of the buffer.

**Figure 5:**
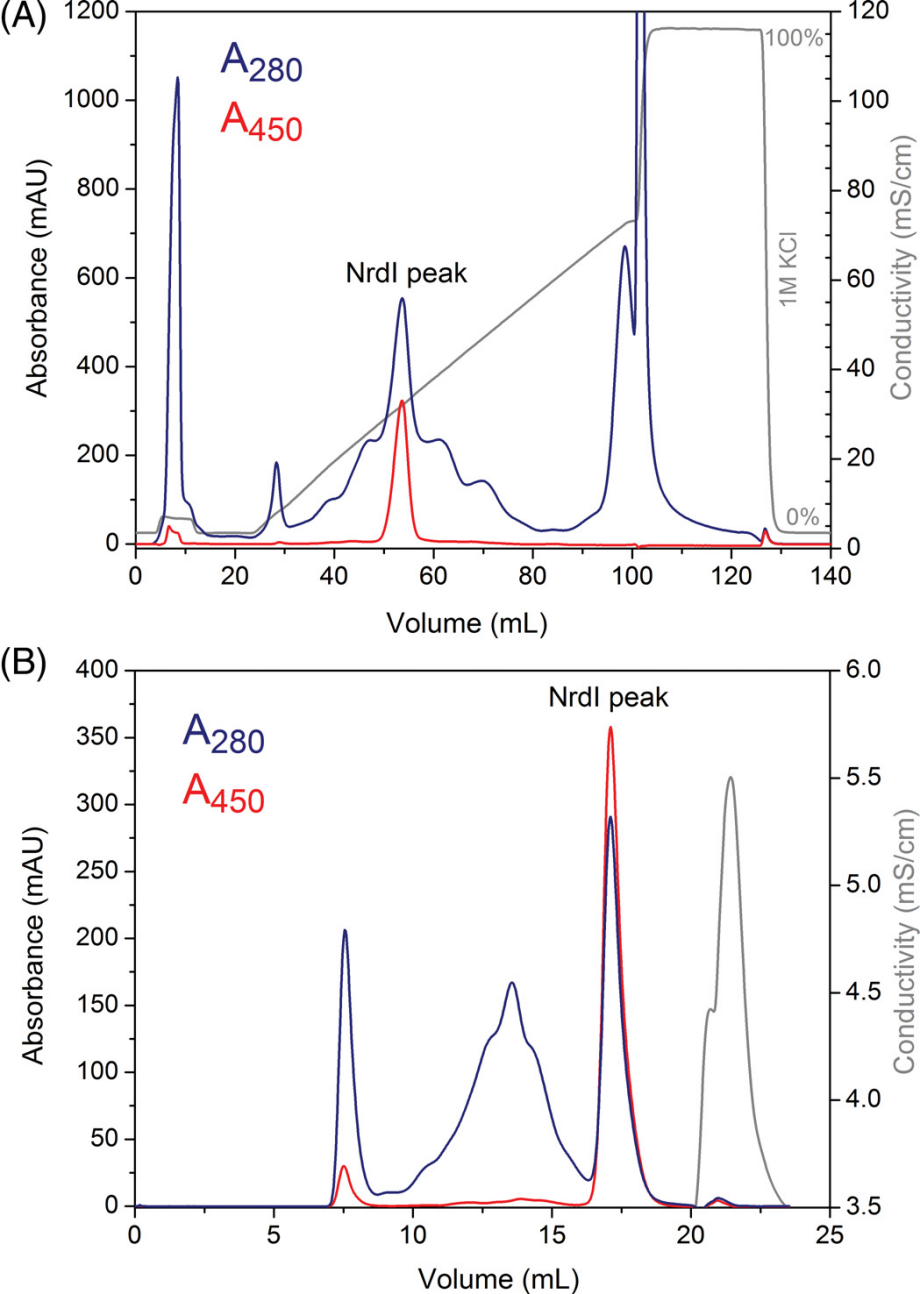
IEX chromatography (A) and GF chromatography (B) of NrdI. (A) IEX of NrdI on a 5 mL HiTrap Q HP column using a 15 CV gradient of 0%–60% 1M KCl. (B) GF of NrdI on a Superdex 200 10/300 GL column. Abs_280nm_ is shown in blue; Abs_450nm_ is shown in red; and conductivity is shown in gray. [Color figure can be viewed at wileyonlinelibrary.com]

The whole volume of the desalted protein from each group is loaded onto the IEX column. Typically, an 80 mL linear gradient of 0%–60% elution buffer is used for protein elution, and the NrdI protein is eluted around 250 mM KCl (Fig. 5A). The NrdI peak is easily distinguishable from remaining proteins in solution due to the strong absorbance at 450 nm. Usually, fractions containing 9 mL protein are pooled and concentrated to a final volume of approximately 300 μL for each group.

For the gel filtration (Fig. 5B), 120 μL of the concentrated, anion‐exchanged sample is loaded onto the column. The remaining protein sample is frozen for succeeding UV–vis experiments. Typically, a peak‐fractionation procedure is chosen to ensure high purity and separation of the pooled fractions. A common volume of 2–4 mL pooled protein is concentrated to approximately 50 μL pure NrdI protein, and frozen for subsequent crystallization experiments. To calculate the total yield of protein, Beer‐Lamberts Law is used. The extinction coefficient for the oxidized, protein‐bound FMN cofactor in NrdI at 450 nm (*ε*_450ox_) is 10.8 mM^−1^cm^−1^ 18. The resulting protein yield varies for different semesters and student groups, averaging at 2–10 mg per L culture medium.

### Part 4: SDS‐PAGE

#### *Background*

SDS‐PAGE is used for inspection of protein expression and protein purity succeeding each purification step.

#### *Experimental*

50 μL 1 x SDS Loading Buffer (NuPAGE® LDS Sample Buffer (4X), Thermo Fisher, Oslo, Norway) is added to each Eppendorf tube containing cells prior to, and after induction, desalted protein sample, IEX fraction, and GF fraction, and mixed well using a pipette. The tubes are heated for 5 minutes on a heat‐block set at 96 °C, vortexed and centrifuged at maximum speed using a benchtop centrifuge. Precast NuPAGE™ Novex™ 4%–12% Bis‐Tris Protein Gels (1.5 mm, 10 wells) (Thermo Fisher) are used to analyze the samples, using a 1% NuPAGE® MES SDS Running Buffer (Thermo Fisher) and a prestained protein ladder (Novex® Sharp Pre‐stained Protein Standard) (Thermo Fisher). The gels are stained with the InstantBlue™Ultrafast Protein Stain (Expedeon) for 15–30 minutes and stored in water for further inspection and photographing for incorporation into student reports.

#### *Results*

**Figure 6:**
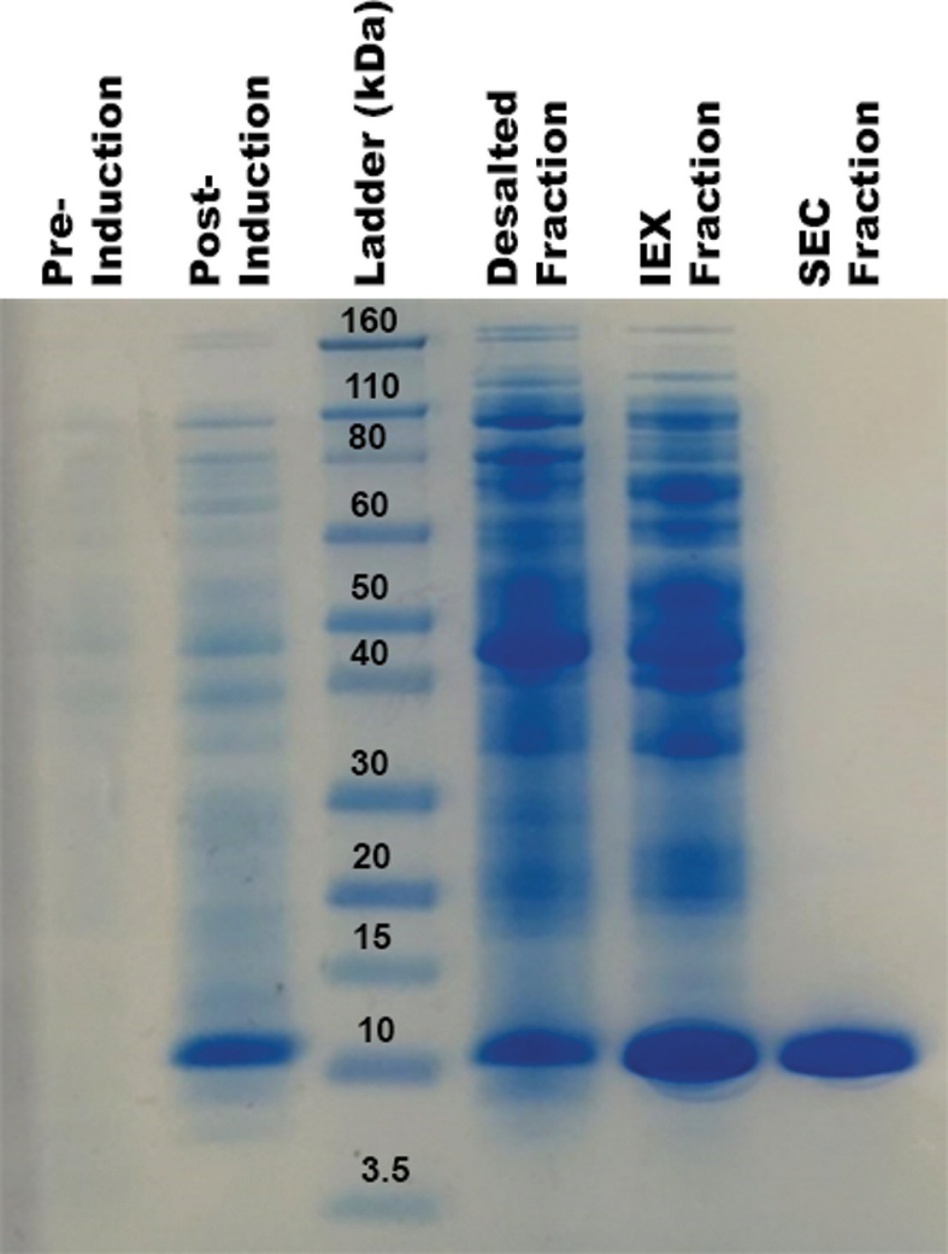
SDS‐PAGE analysis of protein expression and protein purity succeeding various purification steps. From left: Bacterial pellet before induction; bacterial pellet after induction and overexpression for 5 hours; protein ladder (Novex Sharp Protein Standard); protein fraction after protein precipitation with (NH_4_)_2_SO_4_ and desalting on a HiTrap desalting column; protein fraction after IEX on a HiTrap Q HP anion exchange column; protein fraction after SEC on a Superdex 200 10/300 GL gelfiltration column. [Color figure can be viewed at wileyonlinelibrary.com]

To analyze the success of the purification scheme, students perform SDS‐PAGE to examine each step, including protein expression, cell lysis, and purification. A typical SDS‐PAGE gel from a student group is shown in Fig. 6. Protein expression has been successful for all semesters. From inspection of the gels, students can clearly see the improvement in protein purity succeeding each purification step. After the final polishing purification step using SEC, a protein purity of approximately 95% is acchieved, well suited for the following crystallization experiments.

### Part 5: UV–Vis Spectroscopy

#### *Background*

In this course, students are introduced to the principles of spectroscopy, and in particular UV–vis spectroscopy, in addition to basic knowledge in molecular orbital theory. As many reactions in the body involve redox reactions, spectroscopy is an important tool used to investigate and understand aspects of enzymatic reactions.

The NrdI protein is an excellent model for introducing students to the field of UV–vis spectroscopy, as the redox properties of the FMN cofactor can easily be monitored and investigated.

#### *Experimental*

The experiment requires approximtely 1 mL 50 μM NrdI solution. The IEX concentrate (semi‐pure) is used for this exercise. In the presence of oxygen, the NrdI protein exists in its oxidized state. First, the students determine the concentration of their sample, using the *ε*_450ox_ = 10.8 mM^−1^cm^−1^, a UV–vis spectrophotometer, and Beer‐Lamberts Law.

For the investigation of NrdI's redox properties, the students start the experiment by recording a base‐line, using a capped quarts cuvette with degassed 50 mM Tris–HCl, pH = 7.5.

Then, protein is added to the buffer in an appropriate amount, and a spectrum of NrdI with the FMN cofactor in its oxidized state is recorded. 2–10 μL aliquots of a 20 mM sodium dithionite solution (degassed and purged with argon) are added to the sample repeatedly, and mixed, to gradually reduce the FMN cofactor. A spectrum is recorded after each addition, and the spectral changes are monitored and discussed with a teacher. Finally, the results are compared to a colorless protein reference spectrum, for example, 1 mg/mL bovine serum albumin.

#### *Results*

**Figure 7:**
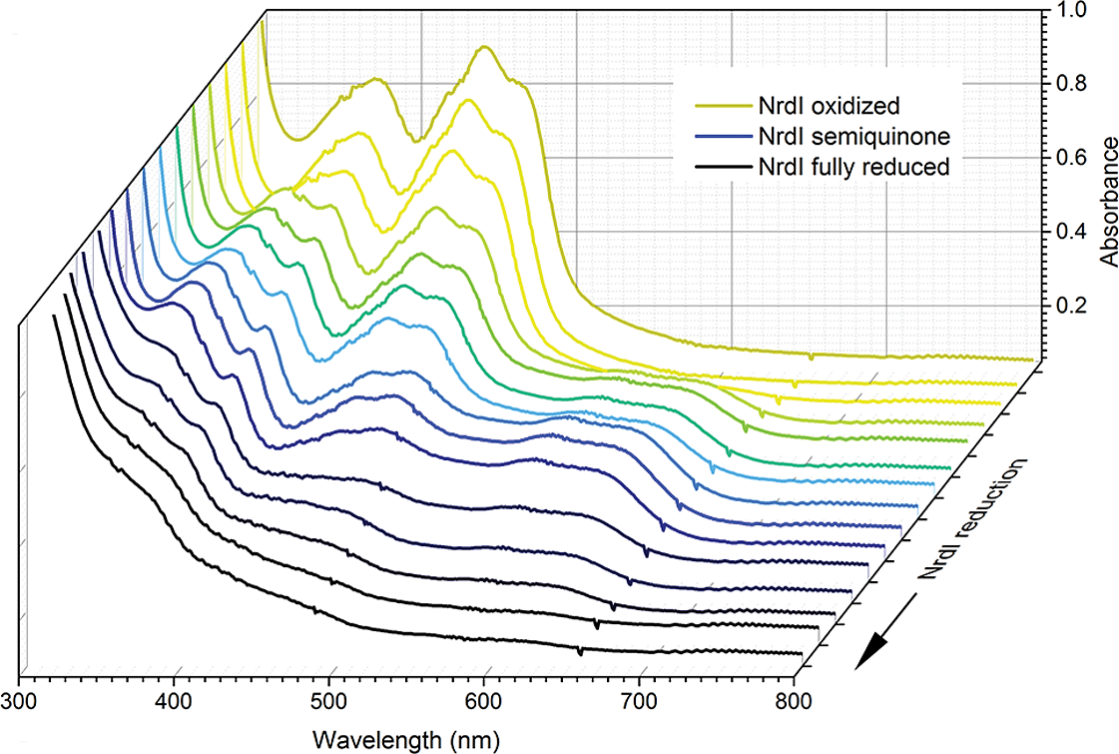
UV–vis absorption spectra of the NrdI protein, titrated with dithionite, showing the reduction of the flavin cofactor; starting from the fully oxidized form (FMN), proceeding through the semiquinone form (FMNH^•^) to the fully reduced hydroquinone form (FMNH_2_). [Color figure can be viewed at wileyonlinelibrary.com]

The time‐resolved UV–vis absorption spectra of the titration of NrdI with dithionite are showed in Fig. 7. It should be noted that the NrdI protein is easily reoxidized by molecular oxygen, so uncapping the cuvette after reduction will result in reoxidation of the protein to the yellow oxidized form. When interpreting the results in Fig. 7, students are asked to consider what happens to the FMN UV–vis spectrum when reductant is added, and how many redox states of the FMN cofactor they can detect. The students are then able to answer one of the two main questions they are asked to answer during the “Gene to Structure and Function” project; namely if NrdI in *B. cereus* can act as a one‐ or two‐electron donor. The buildup of the semiquinone state (Fig. 7) shows that it can function as a one‐electron donor, and therefore, that the two redox potentials E_ox/sq_ and E_sq/hq_ are different.

### Part 6: Protein Crystallization

#### *Background*

The students are introduced to the theory of crystallization, and the techniques and strategies needed to find the crystallization conditions for a protein, and for optimization, in order to obtain crystals suitable for protein crystallography.

Prior to crystallization of the NrdI protein, a crystallization exercise with a model protein (hen‐egg‐white‐lysozyme, Hampton Research) is given as a tutorial (optional) for the students to practice before they set up crystallization drops of their own protein. Crystallization screening has shown that NrdI can be crystallized by the hanging‐drop procedure in a solution of 0.1 M sodium cacodylate, pH 6.5, 0.2 M zinc acetate, and 14% PEG 8000 (precipitant‐solution) 19. To crystallize the purified NrdI protein and optimize the conditions, the students will try to both lower the protein concentration and lower the precipitant concentration to find the best crystallization conditions, giving the largest crystals with highest quality (often indicative by sharp edges and clarity).

#### *Experimental*

The purest, gel‐filtrated NrdI sample is used for the crystallization experiments. Students thaw their protein on ice and spin the samples at 12,000 rpm for 1–2 minutes to remove potential aggregates. Alternatively, the protein sample may be clarified using a 0.22 μm Ultrafree®‐MC (0.5 mL) centrifugal filter unit (Merck Millipore). Next, each group prepares a series of dilutions of the concentrated NrdI protein (1x, 2x, 3x, and 6x dilutions, in 6–24 μL fractions), and the samples are kept on ice. The crystallization drops are set up in a 24‐well culture plate. Prior to setting up the drops on cover slides (Hampton Research), the students grease the plates with silicone using a 5 mL syringe half‐full with silicon grease, with a pipette tip firmly placed on the tip of the syringe. For the crystallization setup, four wells are added 600 μL precipitant‐solution (Wells 1–4), while four wells are added 400 μL precipitant‐solution mixed with 200 μL mqH_2_O (Wells 5–8). Two parallel hanging drops are set up in each of the eight wells by mixing 1.5 μL NrdI solution and 1.5 μL mother liquour, using the four different protein concentrations. The plates are marked with the corresponding group number and stored for about one week prior to inspection.

#### *Results*

**Figure 8:**
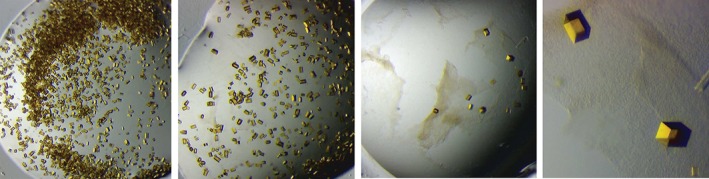
Various examples of NrdI crystals set up by the students in the “From Gene to Structure and Function” module. [Color figure can be viewed at wileyonlinelibrary.com]

The first part of the crystallization laboratory (as described above) is performed during week two of the course. The second part, including inspection of the crystal trays, evaluation of the results, and crystal handling, is set to the last week of the course, enabling crystal growth for approximately one week. Students evaluate the NrdI experiments from the first crystallization laboratory. Each group is provided with a microscope and makes note of the results in a “scoring sheet” (e.g., preciptation, clear drop, crystals, and if crystals size and quality). Figure 8 shows different crystallization drops containing NrdI crystals grown by the students. The NrdI protein is quite easily crystallized, resulting in bright yellow crystals that are easily visualized under a microscope. The students observe the decreasing number of crystals when decreasing precipitant and protein concentration, and in some drops, an increasing size of the crystals. It should be noted for this protein, and for the given crystallization condition, that these crystals normally grow from precipitate, as can be seen from Fig. 8 panel 3, where precipitated protein is still present in the drop. When the crystals grow too fast, indents on each end of the rectangularly shaped NrdI crystals can be observed. The fact that this flavoprotein is yellow in its oxidized form is very convenient for teaching purposes, making this protein a good model for crystallization experiments. In addition to evaluating the results, students get training in crystal handling during the last crystallization laboratory. Each group chooses a drop containing several nice crystals and practice on picking up and moving the crystals to another drop by using a nylon loop. This is how crystals are prepared for crystallographic data collection.

### Part 7: Computer Laboratory—Solving and Investigating the NrdI Structure

#### *Background*

The students are introduced to the theory of protein crystallography through six lectures and six exercises. They learn about the properties of crystals including symmetry, the theory of diffraction, how crystallographic data is collected and processed, how structures are solved, refined, modeled, and validated. They also learn how to interpret crystallographic tables in scientific papers. The focus is for the students to grasp a qualitative understanding of protein crystallography without going into a mathematical description.

**Figure 9:**
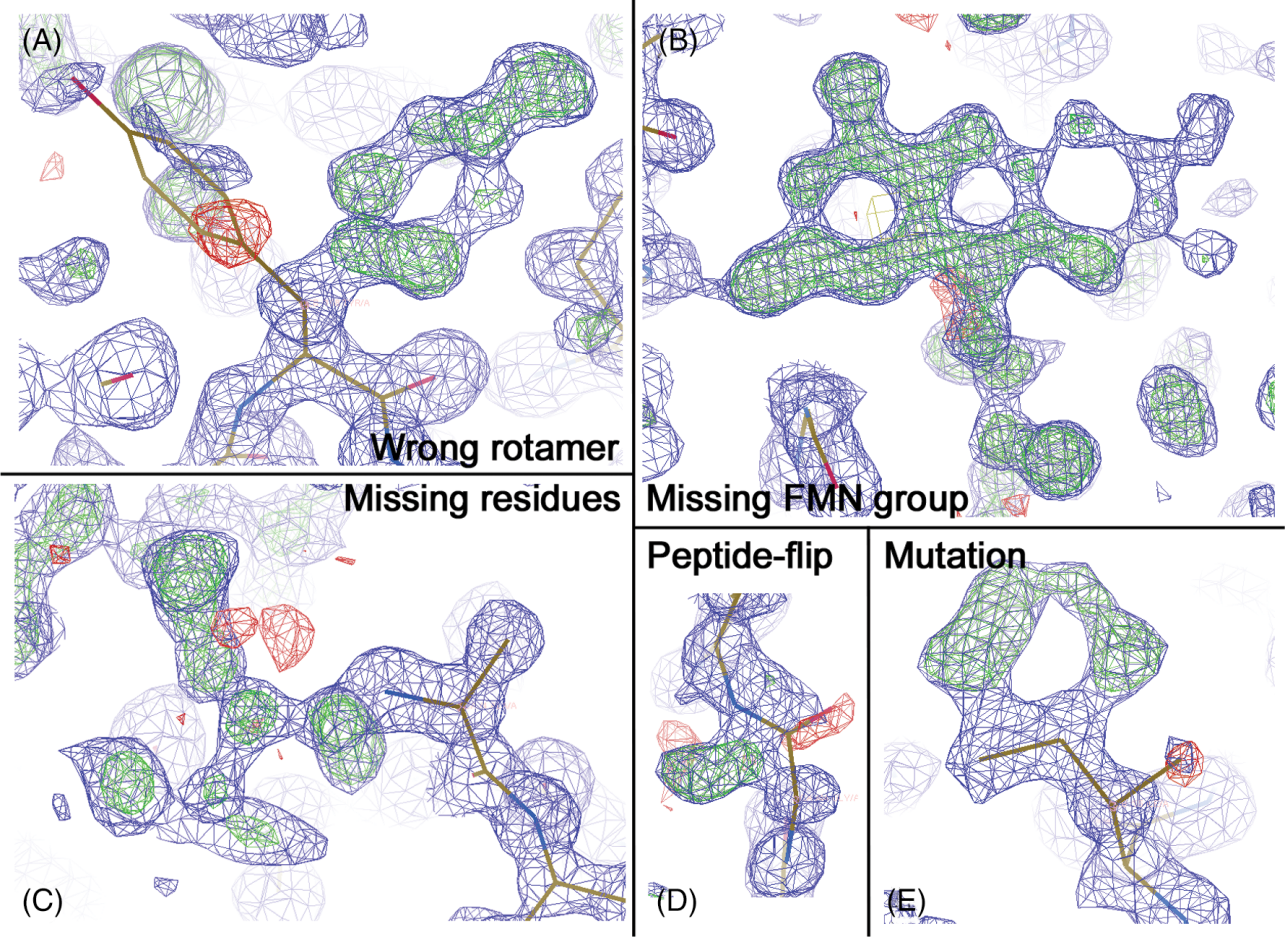
Models of the NrdI structure, showing the corresponding 2F_o_‐F_c_ electron density map (contoured at 1σ in blue) and F_o_‐F_c_ electron density difference maps (contoured at +3σ in green and at ‐3σ in red), illustrating various parts of the model corrected or rebuilt by the students in Coot. (A) Flipping the wrong rotamer of a tyrosine residue. (B) Addition of missing FMN cofactor in electron density. (C) Addition of missing N‐terminal residues (valine‐leucine‐methionine) to the polypeptide chain. (D) Flipping peptide chain with corresponding carbonyl group. (E) Mutating isoleucine to phenylalanine. [Color figure can be viewed at wileyonlinelibrary.com]

**Figure 10:**
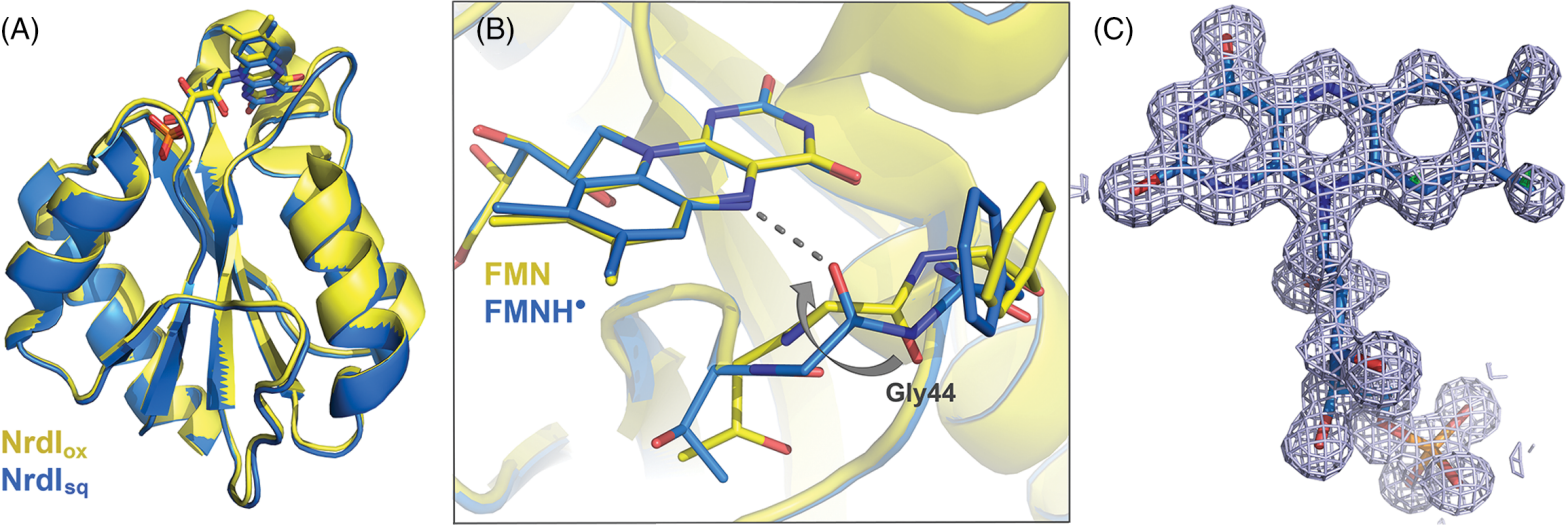
(A) Overall structure of the B. cereus NrdI, with the FMN cofactor represented as sticks. NrdI_ox_ (PDBid:2X2O) is shown in yellow, whereas NrdI_sq_ (PDBid:2X2P) is shown in blue. (B) Structural alignment of NrdI structures with the FMN cofactors in different oxidation states. A conformational change is initiated upon reduction of the cofactor, resulting in a peptide flip orienting the Gly44 carbonyl group toward N5 of the FMN cofactor, and hydrogen bonding (dashed line). (C) The FMN cofactor with corresponding 2F_o_–F_c_ electron density, and F_o_–F_c_ difference maps. [Color figure can be viewed at wileyonlinelibrary.com]

The students crystallize their protein but are not able to travel to the synchrotron to collect X‐ray diffraction data. However, they receive previously collected diffraction images from an NrdI crystal in the semiquinone state. The students will process the data, solve the structure, and do refinement and model buildig in the computer laboratory. The structure is solved by the *molecular replacement* method, where a similar structure is used as a starting model. In order for the students to evaluate errors in the protein chain and adjust their model to the experimental data (generated electron density map), the instructors have introduced alternations to the provided NrdI molecular model. Consequently, the model contains alternations to the peptide chain in the vicinity of the FMN cofactor (as it is generated from the oxidzed structure of NrdI), includes two mutations, three missing N‐terminal residues, a missing FMN group, two side‐chains rotated away from the correct conformations, and missing water molecules (Fig. 9). The students will step‐wise find and model these errors or missing parts. As the start model originates from the oxidized form of NrdI, while the data set the students are working on is the semiquinone state of NrdI, the students should be able to answer one of the two questions they are asked to answer during the “Gene to Structure and Function” project, namely, what changes can be observed around the flavin cofactor when the oxidation state changes. They should observe the peptide‐flip of glycine‐44, where the carbonyl group flips and makes a hydrogen‐bond to the now protonated N(5) of the flavin ring in the semiquinone state (Fig. 10B).

#### *Experimental*

At the computer laboratory, each student works indivdually on a computer. There are different programs available to both process the data and to refine and model the structure. We have chosen to use the CCP4 Software Suite 20, including programs for indexing and integrating data (iMosflm) 21, scaling and merging integrated intensities (AIMLESS) 22, 23, solving structures via molecular replacement (Phaser) 24, and refining the crystal structure (REFMAC) 25. Model building is performed with COOT 26, and structure figures are prepared with PyMOL (Schrödinger, LLC). The NrdI semiquinone X‐ray diffraction data set, the start model and the sequence used in this module is available as online Supporting Information.

##### *Data Collection*

Students are provided with the X‐ray diffraction dataset from the semiquinone NrdI structure on USB sticks that has been collected at the Swiss‐Norwegian Beam‐Line (SNBL) at the European Synchrotron Research Facility in Grenoble, France.

##### *Data Processing*

Each student starts by creating a personal CCP4 project, in order to easily handle all their following files. Indexing of the data is performed using iMosflm. All the diffraction images are loaded, and auto indexing is run. Then, the primitive orthorhombic (oP) crystal lattice should be selected as the best solution (*low penalty* and *high symmetry*), and the mosaicity should have a value between 0.2 and 0.8. Following indexing, cell refinement is performed, and the final unit cell parameters are written down for the student reports. Finally, the data is integrated, and the reulting MTZ file is stored before further processing. Scaling is done in AIMLESS. Students must remember to enable the “*Ensure unique data & add FreeR column*” button, in order to select 5% of the experimental data to be used as a control of the model they will build. Also, data with resolution above 1.15 Å is excluded due to poor quality. After scaling with AIMLESS, the correct space group should be P2_1_2_1_2_1_. Students save the scaling statistics for the reports, including important parameters such as *Overall* and *Outershell* values for *R*_*meas*_, *Mean((I)/sd(I))*, *CC*_1/2_, *Redundancy*, and *Completeness*. Typical scaling statistics are shown in Table 1.

**Table 1:** Typical crystal data quality observed by the students

Space group	P2_1_2_1_2_1_
Cell dimensions (Å)	43.0, 45.5, 56.2
Resolution	27.3–1.15 (1.17–1.15)
*R*_meas_	0.079 (0.667)
Mean *I*/sd(*I*)	10.6 (3.2)
*CC*_1/2_	0.989 (0.780)
Completeness (%)	99.3 (100)
Redundancy	3.8 (3.8)

The values in parenthesis are for the highest resolution shell.

##### *Phasing Using Molecular Replacement*

The crystal structure is solved by molecular replacement using the program *Phaser*. Phaser is run using three necessary files; the MTZ file from AIMLESS, the coordinate file including the starting model, and the text file including the NrdI amino acid sequence. The sequence identity is assumed to be 100%. The two latter files are provided to each student on a USB‐stick.

##### *Modeling and Refinement*

When the structure is solved, the students start an iterative process of modeling and structure refinement. This is performed using Coot and REFMAC, respectively. First, an initial refinement is performed to obtain a rough model and electron density map, by running 10 cycles of restrained refinement using isotropic B‐factors. The final *R‐factor* after the first refinement should be 30%–35%. The students write down the *R‐factor* and *Rfree* values for each refinement step (Table 2) and document changes they make to their model during model building. The first step of model building is to add the missing FMN cofactor to the model (Fig. 9B). The easiest way to add the FMN cofactor is to start by locating unmodeled electron density in Coot (using the *Validate* tool). When the students have recognized the shape of the FMN electron density, the model of the FMN cofactor is imported into the elctron density map. Hydrogen atoms are removed from the FMN model, and the cofactor is fit into the unoccupied density, using the *Rotate Translate Zoom* button, and the *Real‐Space Refinement* tool. Prior to a new refinement cycle, the new FMN model is merged together with the protein PDB file. In the second modeling step, the students go through all the residues to investigate the amino acid chain geometries improving the protein model. They should find two amino acid residues that have been mutated (Fig. 9E) in the starting model, two side chains that have a wrong conformation (rotamer) (Fig. 9A), and a peptide‐flip (Fig. 9D) by using the buttons *Simple Mutate*, *Rotamers*, and *Flip Peptide*. In the third modeling step, the students need to build in three N‐terminal amino acids that are missing from the model by using *Add Terminal Residue* and *Simple Mutate* tools (Fig. 9C). As an option, students can also add a zinc ion and a cacodylate ion, which are found in the electron density. In the fourth modeling step, water molecules are added using the *Find Waters* tool in Coot, and a final refinement is performed with REFMAC. The final model is validated in the end, using tools found in Coot, such as the *Ramachandran Plot* and the *Rotamer* and *Density Fit* analyses. Next, the electron density maps are exported from CCP4 for further use in PyMOL. This is done using the *Map & Mask Utilities* menu in CCP4, where the 2F_0_‐F_c_ map is exported as an _FWT.map file, and the F_0_‐F_c_ difference map is exported as a _DELFWT.map file. Both files are given the extension .ccp4 prior to loading them into PyMOL.

**Table 2:** Typical R‐factor and R_free_ values after the different modeling and refinement steps performed by the students

After	*R‐factor*	*R*_free_
Molecular replacement	0.379	0.400
Initial refinement	0.338	0.352
Addition of the FMN cofactor	0.317	0.328
Correction of mutations/rotamers	0.305	0.317
Addition of missing N‐terminal residues	0.296	0.309
Addition of water molecules	0.270	0.289

##### *Making Structure Figures in PyMOL*

In the first part of the PyMOL‐laboratory, an introduction to the program is given as a PyMOL tutorial on a model protein (optional). In the second part, students use their newly solved NrdI structures and prepare figures for their reports. For the students to learn more about interpreting protein structures, we have added additional computer laboratory exercises where the students use PyMOL to study binding of drugs to proteins, protein–protein interactions, and describe protein properties (optional).

#### *Results*

The results from the computer laboratory are included in the articles, including three figures of the NrdI protein that are made individually by each student; an overall structure of the protein including the FMN cofactor (cartoon and sticks, respectively), an image zoomed in on the FMN cofactor showing the cofactor with the corresponding electron density maps made in CCP4 (total density and difference densities), and an overlay of the students' NrdI_sq_ protein structure with the provided NrdI_ox_ structure, zoomed in on the FMN cofactor (see Fig. 10). The latter figure illustrates the difference in the glycine‐44 conformation in the structures of NrdI_ox_ and NrdI_sq_, revealing the hydrogen bond to the FMN cofactor as a result of the change in redox state in the two crystals.

## Student Reports

The students write and hand‐in one individual article as a report from the whole “From Gene to Structure and Funciton” project. By doing this, the students get a unique overview of the whole course and can see the different methods in context. The article is written in the American Chemical Society journal template, with an Abstract, Introduction, Materials and Methods, Results and Discussion part. The goal is for the students to practice scientific writing and to be able to present their work in a short, concise, and precise way. The article is suggested to be around 2000 words.

## Student Evaluation

The student feedback on this course has been very positive, and the original goals set out when this course was redesigned seems to have been achieved. In Table 3, selected evaluation questions from the Fall 2016 are shown. The overall score for the module is 4.9 out of 5.0. The students further give high ratings for the learning outcome, the one‐project idea, higher engagement, more research relevance, and the depth and breadth of the course. They have also gained more confidence to use these methods themselves. When the students are asked what generic skills they have learned through the course, they point out academic writing, interacting with students and teachers, team work, and time management. These are important skills for the students when they are continuing their work with a master thesis in a research group. Several of the above mentioned outcomes could also coincide with important goals in CUREs‐based courses. Regarding the gained scientific skills, stundents acknowledge learning different techniques and being aware of various applications of the methods learned.

**Table 3:** Student feedback (Rate on a scale 1–5, where 5 is best/highest score). Evaluation of the module given during Fall 2016, answered by 23 out of 30 students anonymously online

Question	Score
How would you rate the module?	4.9
How would you rate your learning outcome?	4.6
How would you rate handing‐in the report as an article in an article template?	4.5
How do you rate running this part as one large project “From Gene to Structure and Function”, compared to laboratory courses with smaller, separated laboratory exercises instead?	4.5
How engaging was it to run one project compared to more separated laboratory exercises?	4.6
Do you feel this project gave you more insight into research than normal laboratory courses?	4.6
How confident are you in using these methods/topics yourself in the future?	4.0
How well do you feel going in depth in one example protein worked out with respect to opening up and extending for future use of related/similar methods/system, the so‐called breadth?	4.7

## Scheduling the Course over a Whole Semester

Although running the course in the 2.5 week format enhances the research focus and allows the students to fully dwell into the project on a day‐to‐day basis, it is also possible to run it as a one‐session‐a‐week course during a whole semester. For a 10–14 weeks course, a proposed timescale of the laboratory part is presented in Table 4. As the duration of the various laboratory days will vary, any spare time in between experiments could be filled with lectures and exercises. Alternatively, the first weeks of the course could exclusively be comprised of lectures, before introducing the practical part. Furthermore, a semester‐based course could also allow for separate weeks of experimental optimization, allowing students to participate in experimental design and perform small‐scale optimization experiments prior to proceeding with the most successful protocol.

**Table 4:** Suggested schedule of the laboratory part for a one‐day‐per‐week course

Week #	Experimental procedure	Estimated time per student/group^a^
1	Protein expression and cell harvesting^b^	8 hr^c^
2	Cell lysis and protein extraction	8 hr
3	Protein chromatography^d^	3 hr
4	SDS‐PAGE	2 hr
5	Protein crystallization	1–2 hr
6	Computer laboratory, Day 1	8 hr
7	Computer laboratory, Day 2	4 hr
8	Inspection of crystals and UV–vis spectroscopy	2 hr

a Experiments in weeks 1, 2, 5, 6, and 7 are run in whole classes/groups in parallell, and the estimated time usually corresponds to the time spent by all students in total. Experiments in weeks 3, 4, and 8 require more specialized instruments, and the total time will depend on available instruments at a given facility.

b Overnight cultures should be started by the TAs on the preceding day.

c Inoculation of day cultures, induction, and cell harvesting is performed at different times of the day, leaving space for other activities in between experiments.

d If available instrumentation is limited, an extra chromatography session could be included.

## Further Extensions of the Course

The course could be expanded by extending the time frame of the course, and with increased access to resources such as staff, instrumentation, and biological and biophysical expertise. By extending the length of the course, the students could be included in the experimental design process, resulting in a more discovery‐based laboratory course in a real‐world research setting. Small‐scale optimization experiments could be performed by the students, allowing the different groups to test for example, various protein expression conditions, ammonium‐sulfate concentrations for protein precipitation, and optimization of the IEX chromatography procedure.

By including additional biochemical methods such as cloning, mutagenesis, nuclear magnetic resonance (NMR) spectroscopy, mass spectrometry (MS), and various protein–protein interaction techniques, the students' laboratory skills could be further improved and broadened. A topic that could be included is recombinant DNA technology, involving cloning of the *NrdI* gene into a suitable vector by the students. Student participation in experimental design could be further increased by giving the students the opportunity to introduce mutations, based on their interpretation of the literature in respect to for example, the relative role of a set of chosen residues` effect on cofactor binding, stability, binding to redox‐partners, and so forth. Moreover, students could be encouraged to design an experimental procedure in order to test hypotheses regarding for example cloning vector choice, expression variables, buffer choice, and chromatographic procedures. Protein identity could be analyzed through MS. The “From Gene to Structure and Function” module could also be extended to include further functional studies of the NrdI protein. As the flavodoxin‐like protein has been shown to be reduced by various ferredoxin/flavodoxin NADP^+^ oxidoreductases (FNRs) 27, investigations of redox partners able to provide reducing equivalents to NrdI could be performed through kinetic studies. The specific interaction between NrdI and its redox partners, the FNRs, as well as the RNR class Ib small subunit NrdF, can be investigated through studies of protein–protein interactions using microscale thermophoresis or isothermal titration calorimetry. By extending the course, additional and complementary biochemical techniques could be included in this course, enhancing the students' scientific knowledge, in addition to the incorporation of more student‐centered, active learning styles.

As described in the introduction, related research‐based biochemistry laboratory courses have been published, but they differ both with respect to topics and required skills. Researchers at the University of Nebraska have developed a nice intensive research‐based course with a similar focus and layout as our module, starting from cell transformation, and ending with protein crystallization 4. However, the latter has a larger focus on protein purification, while our course instead includes the additional part of solving the crystal structure, as well as spectroscopic characterization. Several nice CUREs‐based biochemistry courses have been published during recent years. One example is the course from Haverford College in Pennsylvania, including hypothesis generation, writing grant proposals, group meetings, team work, open laboratory and possibility for several interdisciplinary directions 15. These courses cover an important niche in the undergraduate education but are often quite faculty/TA resource demanding. Our course is more closely guided, less resource demaning, and can be run for larger student groups, and still maintains a project‐based and discovery‐driven approach. Additonally, compared to most other courses, it focuses on writing up the project as a research paper, which is an important skill for the students to learn. Nevertheless, a similar biochemistry course to the one described at Haverford College would be a nice complementary and follow‐up course for the students.

## Summary and Conclusions

The “From Gene to Structure and Function” module is given as an intensive 2.5‐week full‐day course, including experimental laboratory assignments, computer laboratory, lectures, and classroom exercises. The module covers important topics within the fields of biochemistry, molecular biology, and structural biology, meant to give students the confidence and competence needed for future work in molecular bioscience research. These topics include practical and theoretical knowledge in protein expression, protein purification techniques, UV–vis spectroscopy, protein crystallization and crystallography. The module is designed as an actual, continuous research project, as opposed to most laboratory courses, often containing separate laboratory tasks and topics. The research‐like environment provided for the students give rise to a higher degree of commitment and engagement, because students are allowed to work on a given enzyme system and one certain protein, which they will overexpress, purify, crystallize, and characterize biochemically during the course. The NrdI protein system used here has shown to be an excellent and successfull model protein for this type of course. The course is well‐suited as a late undergraduate, as well as a master course, and has been thoroughly optimized during the several years it has been taught. This has resulted in excellent student feedback. The course provides important scientific and generic skills and experience for the students and prepare and give them confidence in future work as researchers. This module should be adaptable and adjustable to be used at different institutions.

## Conflict of Interest

The authors declare no conflict of interest.

## Supporting information

Appendix S1 Supporting information
